# Calpain mobilizes Atg9/Bif-1 vesicles from Golgi stacks upon autophagy induction by thapsigargin

**DOI:** 10.1242/bio.022806

**Published:** 2017-03-16

**Authors:** Elena Marcassa, Marzia Raimondi, Tahira Anwar, Eeva-Liisa Eskelinen, Michael P. Myers, Gianluca Triolo, Claudio Schneider, Francesca Demarchi

**Affiliations:** 1 C.I.B. National Laboratory, AREA Science Park, Padriciano 99, Trieste 34149, Italy; 2 Department of Biosciences, University of Helsinki, PO Box 56, Helsinki 00014, Finland; 3 International Centre for Genetic Engineering and Biotechnology, AREA Science Park − Padriciano 99, Trieste 34149, Italy

**Keywords:** Calpain, CAPNS1, Bif-1, Endophilin B1, Autophagy, Thapsigargin

## Abstract

CAPNS1 is essential for stability and function of the ubiquitous calcium-dependent proteases micro- and milli-calpain. Upon inhibition of the endoplasmic reticulum Ca^2+^ ATPase by 100 nM thapsigargin, both micro-calpain and autophagy are activated in human U2OS osteosarcoma cells in a CAPNS1-dependent manner. As reported for other autophagy triggers, thapsigargin treatment induces Golgi fragmentation and fusion of Atg9/Bif-1-containing vesicles with LC3 bodies in control cells. By contrast, CAPNS1 depletion is coupled with an accumulation of LC3 bodies and Rab5 early endosomes. Moreover, Atg9 and Bif-1 remain in the GM130-positive Golgi stacks and Atg9 fails to interact with the endocytic route marker transferrin receptor and with the core autophagic protein Vps34 in CAPNS1-depleted cells. Ectopic expression of a Bif-1 point mutant resistant to calpain processing is coupled to endogenous p62 and LC3-II accumulation. Altogether, these data indicate that calpain allows dynamic flux of Atg9/Bif-1 vesicles from the Golgi toward the budding autophagosome.

## Abstract (precis)

**Summary:** ER stress triggers calpain-dependent Bif-1 activation and induction of autophagosome maturation by promoting ATG9/Bif-1 vesicle trafficking and fusion with LC3 bodies.

## INTRODUCTION

The ubiquitous μ- and m-calpain are calcium-dependent neutral cysteine proteases composed of an 80 kDa catalytic subunit, CAPN1 or CAPN2, respectively, and a common 28 kDa regulatory subunit, CAPNS1 (Goll et al., 2003). Targeted disruption of CAPNS1 results in embryonic lethality at day 10 post-conception, as a consequence of severe defects in vascular development (Arthur et al., 2000). Calpains proteolytically process a number of specific substrates, in a tightly regulated manner, and therefore exert pleiotropic functions within the living cell. For example, they modulate the adhesive complex dynamics in adherent cells (Bhatt et al., 2002), exerting both positive and negative functions in cellular adhesion and movement. Similarly, calpain can positively regulate autophagy (Demarchi et al., 2006; Yoon et al., 2008; Escalante et al., 2013) and switch it off (Yousefi et al., 2006; Menzies et al., 2015). Interestingly, autophagy is also involved in the modulation of cellular movements (Tuloup-Minguez et al., 2013; Galavotti et al., 2013).

Ubiquitous calpains are associated with the endoplasmic reticulum and Golgi apparatus, both proposed as sites for autophagosome nucleation (Lamb et al., 2013). A number of environmental stimuli determine endoplasmic reticulum stress, and consequently induce autophagy (Ogata et al., 2006; Ding et al., 2007) and trigger calpain activation. In particular, the sarco/endoplasmic reticulum Ca^2+^ ATPase (SERCA) is inhibited by thapsigargin in the nanomolar range, with consequent release of calcium from the endoplasmic reticulum coupled to calpain activation (Martinez et al., 2010) and autophagy initiation (Ogata et al., 2006). By contrast, micromolar concentrations of thapsigargin inhibit rises in intracellular calcium (Geiszt et al., 1995). Accordingly, 3 µM levels of thapsigargin lead to accumulation of mature autophagosomes by blocking autophagosomes fusion with the endocytic system (Ganley et al., 2011). The transmembrane protein Atg9 resides in the trans-Golgi network and late endosomes, and upon autophagy induction, it redistributes to peripheral cytoplasm where it co-localizes with LC3, in an ULK1-dependent manner(Yang et al., 2006). Moreover, Bax-interacting factor 1 Bif-1/Endophilin B1 promotes fission of Atg9-positive Golgi membranes and their trafficking towards the site of autophagosomes formation (Takahashi et al., 2011). Bif-1 is characterized by a Bin Amphiphysin Rvs (N-BAR) domain, necessary for binding to and curvature of the double lipid layer, and a C-terminal Src-homology 3 (SH3) domain, that allows interaction with proline-rich proteins (Pierrat et al., 2001). In particular, Bif-1 binding to UVRAG (ultraviolet irradiation resistant-associated gene) protein mediates Beclin1 recruitment to the phagophore and, as a consequence, activation of autophagy (Takahashi et al., 2007).

We previously demonstrated the requirement of CAPNS1 for autophagosome formation in response to rapamycin in MEFs and human osteosarcoma U2OS cells. In this study, we found involvement of CAPNS1 in autophagy modulation in response to thapsigargin and identified Bif-1 cleavage by calpain as a possible mechanism for regulation of the early stages of autophagy by calpain. CAPNS1 depletion is coupled to a clear alteration in the distribution of the Golgi stacks, and a deregulation in Atg9-Bif-1 dynamics upon autophagy induction by thapsigargin.

## RESULTS

### Thapsigargin triggers calpain activation and autophagy in human U2OS cells

We previously showed that CAPNS1 depleted human osteosarcoma U2OS cells and CAPNS1^−/−^ MEFs fail to induce autophagosomes formation in response to nutrient deprivation and rapamycin, the classic autophagy*-*inducing stimuli*.* (Demarchi et al., 2006). In order to identify the molecular basis of this impairment, we compared several steps of autophagosome formation in CAPNS1-depleted and control U2OS cells. As an autophagy trigger, we used 100 nM thapsigargin, since this drug targets the endoplasmic reticulum, where calpain resides and autophagosomes originate. In addition, thapsigargin was reported to activate both calpain and autophagy in various systems when used in the nanomolar range.

**Fig. 1.**
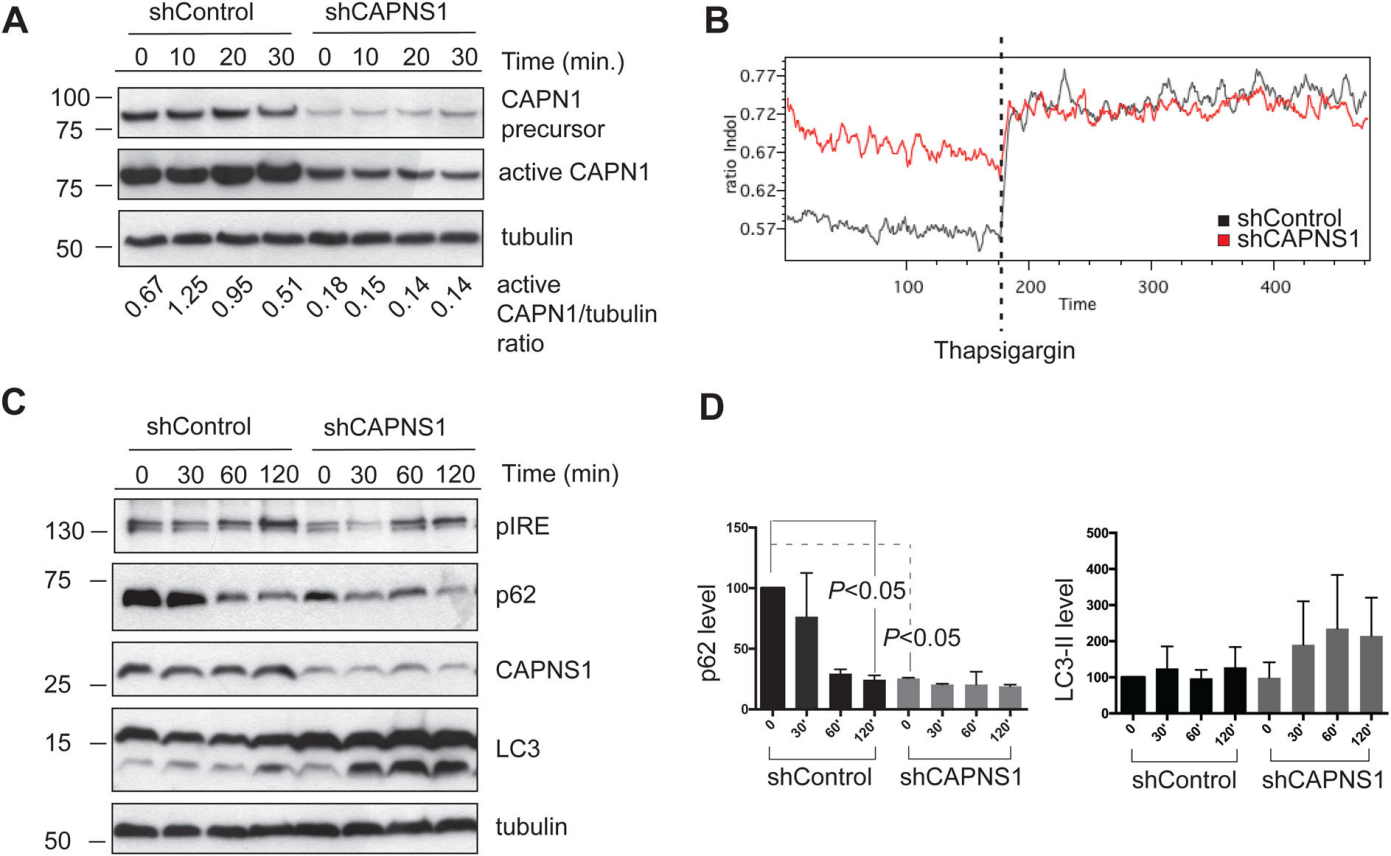
**Thapsigargin treatment induces calpain activation and autophagy.** (A) Control and shCAPNS1 cells were treated with 100 nM thapsigargin for 10, 20 and 30 min and the lysates subjected to western blot analysis to quantify the precursor and active form of CAPN1. The ratio between active CAPN1 and tubulin is reported below each lane. (B) Time course measurement of intracellular calcium concentration. The plot indicates the ratio of mean Indo Violet/Indo Blue emission values of each cell population at individual time points (minutes). The ratio corresponds to the relative calcium concentration of control and shCAPNS1 U2OS cells before and after addition of thapsigargin (100 nm final). Moving average was used as smoothing method. (C) shCAPNS1 cells were treated with 100 nM thapsigargin for 30, 60 and 120 min and the lysates subjected to western blot analysis to detect LC3, p62, pIRE, CAPNS1 and tubulin. (D) Levels of p62 and LC3 normalized to tubulin levels.

In order to evaluate calpain activation, 100 nM thapsigargin was added to control and shCAPNS1 U2OS cells and lysates were collected at the following time points: 0, 10, 20, and 30 min. As shown in the western blot of Fig. 1A, thapsigargin can rapidly induce a reduction in CAPN1 precursor, coupled to an increase in active CAPN1. As expected, CAPN1 protein levels are sharply reduced in CAPNS1-depleted cells (Arthur et al., 2000). The increase of intracellular calcium after thapsigargin addition was verified using Indo1-AM, a ratiometric calcium probe, and FACS analysis. A representative experiment is shown in Fig. 1B. Thapsigargin triggers an increase of cytoplasmic calcium in both control and shCAPNS1 U2OS cells. Notably, basal calcium level is higher in CAPNS1-depleted cells with respect to control cells. A similar phenotype was recently reported for CAPN3-depleted muscle cells (Toral-Ojeda et al., 2016).

In order to monitor autophagosome formation kinetics, control and shCAPNS1 U2OS cells were incubated with 100 nM thapsigargin for 0, 30, 60, 120 min. Next, the cell lysates were collected and utilized for immunoblotting analysis of autophagy markers (Fig. 1C). In control cells, 120 min after thapsigargin addition, LC3-I is converted in its lipidated form LC3-II, while in shCAPNS1 cells the kinetics of LC3 lipidation appears faster and both LC3 forms accumulate. As expected for autophagy-competent cells, p62 degradation couples LC3 lipidation in control cells. On the contrary, in shCAPNS1 cells, p62 levels remain almost stable, thus suggesting the existence of a block in autophagic clearance (Fig. 1D). The basal levels of p62 are lower in shCAPNS1 cells compared with control cells. This might be due to an adaptation of the cells to cope with the clearance defect. As a control for thapsigargin treatment efficacy, we checked the phosphorylation of the inositol-requiring kinase pIRE1 one of the effectors of the unfolded protein response (UPR). Collectively, the data indicate that CAPNS1 depletion perturbs autophagosome clearance in response to thapsigargin treatment.

### CAPNS1 depletion is coupled to an accumulation of LC3-II-positive structures

**Fig. 2.**
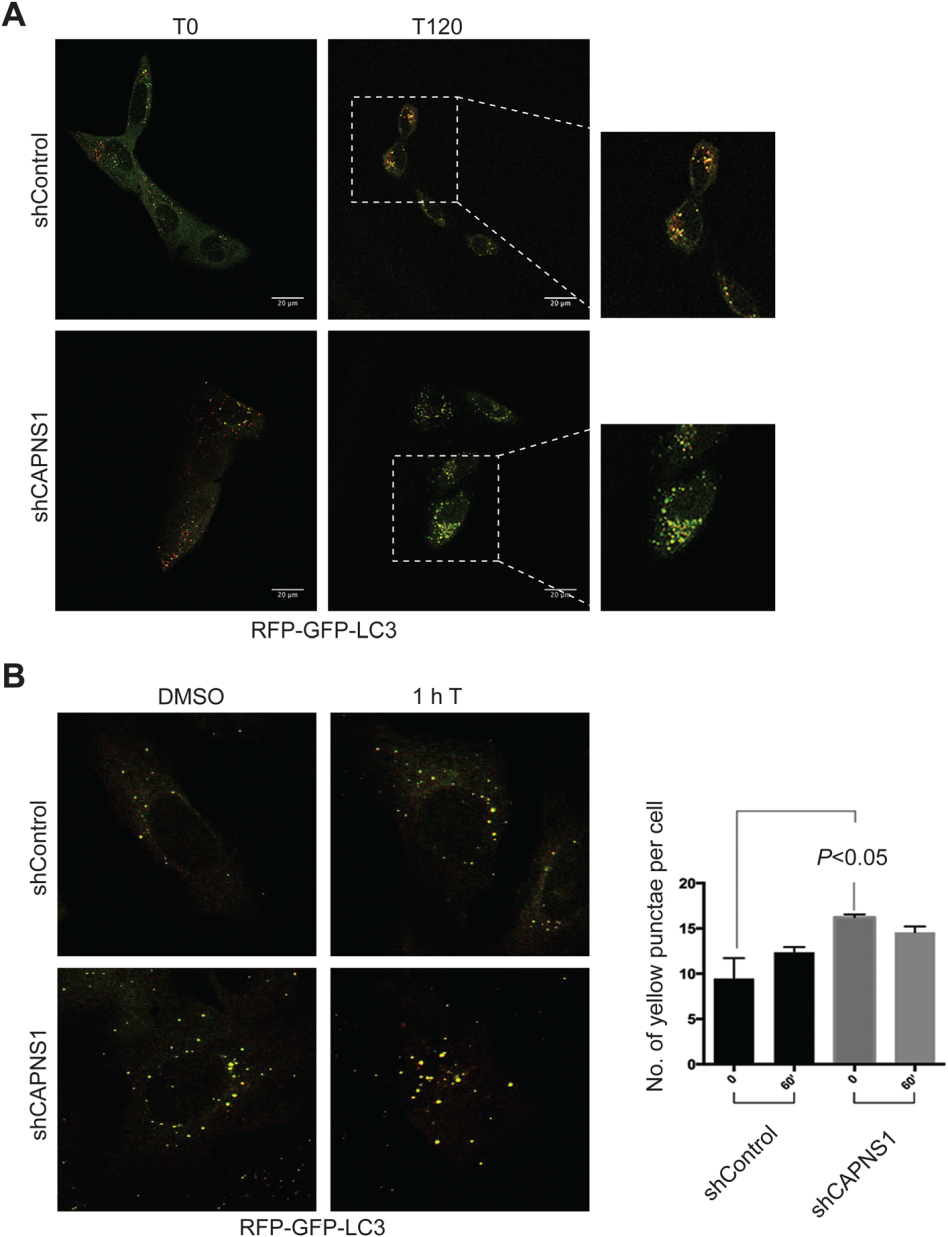
**CAPNS1 depletion causes accumulation of LC3-positive structures.** (A) Control and CAPNS1-depleted U2OS cells were treated with RFP-GFP-LC3 baculovirus reagent. 24 h later, the cells were analysed under a confocal microscope over a 120 min time-lapse experiment. After the first 15 min, 100 nM thapsigargin was added to induce autophagy. The first (T0) and last images (T120) of the experiments are shown. (B) Control and CAPNS1-depleted U2OS cells were treated with commercial RFP-GFP-LC3-expressing baculovirus. 24 h later, the cells were treated with 100 nM thapsigargin or DMSO as control, for 1 h, and then fixed and analysed. Representative confocal microscopy pictures are shown. At least 25 cells were analysed for each replica and the number of yellow dots per cell was counted. The graph reports the mean and s.d. calculated for three independent experiments.

In order to further characterize the effect of calpain on the dynamics of LC3 bodies, live-cell imaging experiments were performed. In particular, we monitored RFP-GFP-LC3 bodies upon thapsigargin treatment, both in control U2OS cells and in CAPNS1-depleted U2OS cells. RFP-GFP-LC3 bodies appear as yellow dots. These structures appear as red dots after their fusion with the lysosomes, due to the acidification that bleaches the GFP fluorescence. U2OS cells were seeded on plates and grown for 24 h; then a commercial reagent designed for RFP-GFP-LC3 expression was added to the cells. 24 h later, RFP-GFP-LC3 bodies were analysed in a 120 min time-lapse experiment; 100 nM thapsigargin was added to the cells after the first 15 min of acquisition. In CAPNS1-depleted cells, we noticed a progressive increase in the level of yellow dots as compared with control cells. This increase is evident in Fig. 2A, which shows the first and last images of representative time-lapse experiments, included as Movies 1,2. In order to quantify the accumulation of RFP-GFP-LC3 bodies, the cells were fixed after 2 h of incubation with 100 nM thapsigargin. Fig. 2B shows representative fields and the average number of yellow dots counted in three independent experiments. CAPNS1 depletion is coupled to an increase in the number of yellow dots, in accordance with the increase in LC3-II shown in Fig. 1C. After treatment with thapsigargin, the number of yellow dots per cell is similar in control and CAPNS1-depleted cells. However, the dots are larger in CAPNS1-depleted cells, as evident in the representative pictures of Fig. 2B and in the time-lapse Movies 1,2. This result is consistent with the increase in LC3-II measured by quantification of western blots data shown in Fig. 1C.

### Early endosome dynamics is perturbed in CAPNS1-depleted cells

**Fig. 3.**
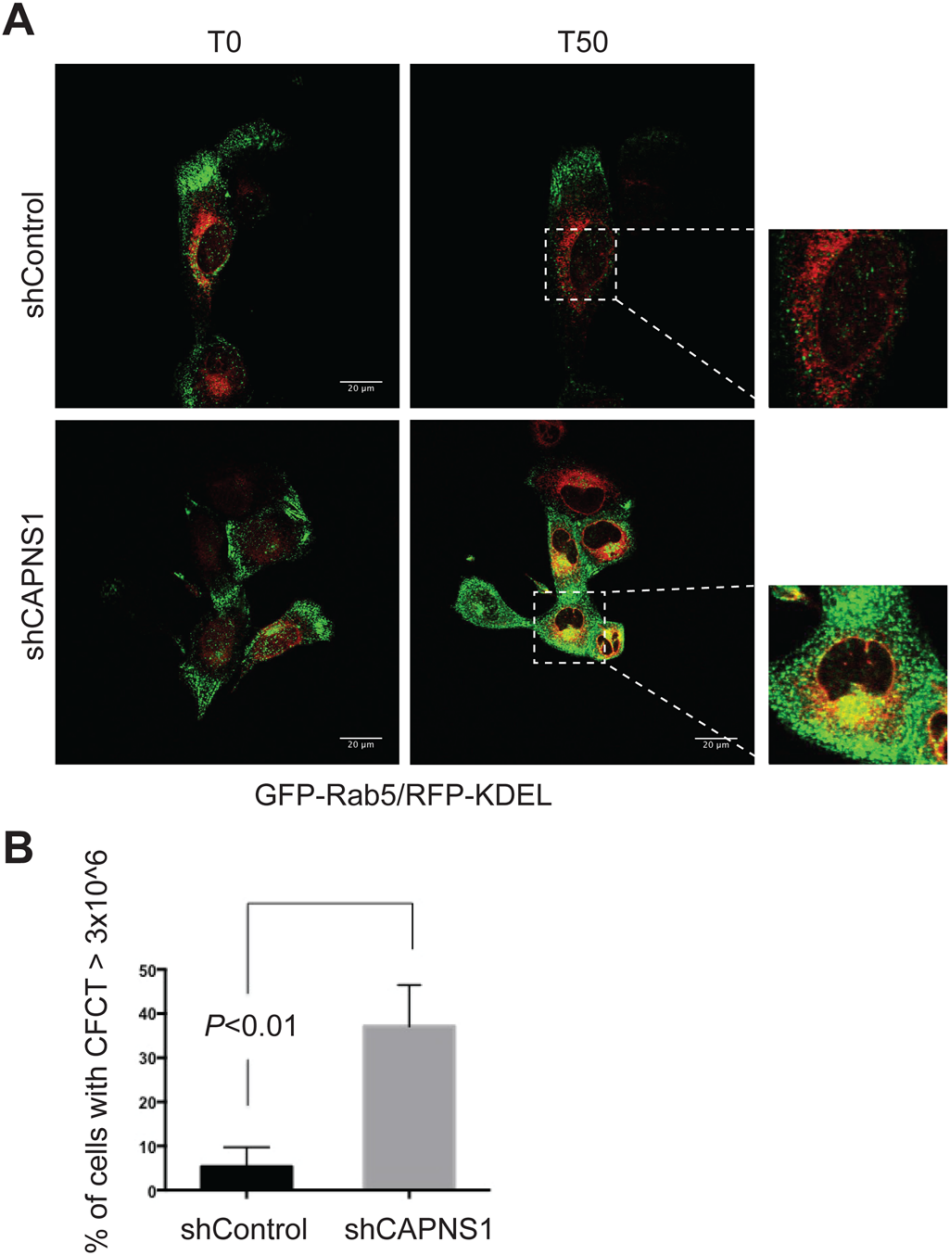
**Early endosome dynamics is impaired in CAPNS1-depleted cells.** (A) Control and shCAPNS1 U2OS cells were grown on Petri dishes, treated with commercial baculovirus reagent expressing GFP-Rab5 protein and RFP-KDEL. 24 h later, the cells were analysed using a confocal microscope to detect the dynamics of Rab5-positive vesicles. Images were acquired every 2 min over a period of 50 min. The first (T0) and last (T50) pictures of each-time lapse experiment are shown. Scale bars: 20 μm. (B) Corrected total cell fluorescence (CTCF) levels of GFP-Rab5 measured on 100 fixed control and shCAPNS1 cells. The graph indicates the percentage of cells that show a CTFC ≥3×10^6^; the error bars represent s.d. of three different independent experiments; *P*<0.01.

A large body of published data indicate the involvement of early endosomes in the process of autophagosome formation (Puri et al., 2013; Ao et al., 2014). We previously reported that CAPNS1 depletion causes impairment in autophagosomes formation and ectopic LC3 accumulation in endosome-like vesicles (Demarchi et al., 2006). As reported above, we found that CAPNS1 depletion influences RFP-GFP-LC3 trafficking in thapsigargin-treated cells. Therefore, we expected a similar behaviour for Rab5-positive endosomes. To verify this hypothesis, we applied the same videomicroscopy technology to monitor early endosome behaviour upon thapsigargin treatment in live cells. Control and CAPNS1-depleted U2OS cells were treated with two commercial baculoviruses expressing the early endosome marker GFP-Rab5 and the endoplasmic reticulum marker KDEL-calreticulin. 24 h later, a 50 min time-lapse experiment was performed using a confocal microscope. After the first 15 min of image acquisition, 100 nM thapsigargin was added to trigger calpain activation and induce autophagy. In both cell lines, labelled endosomes move from the periphery of the cell toward the endoplasmic reticulum (Movies 3,4). Notably, in shCAPNS1 cells, stained endosomes accumulate and gather in a perinuclear region, (Fig. 3A, bottom panels). The accumulation is not observed in control cells (Fig. 3A, top panels). In order to further verify Rab5 behaviour and to quantify this phenotype, the same experiment was repeated and the cells were analysed after fixation on a coverslip. Corrected total cell fluorescence (CTCF) was measured using ImageJ software. As indicated in the graph of Fig. 3B, 1 h after thapsigargin addition, almost 40% of CAPNS1-depleted cells are characterized by a CTFC ≥3×10^6^, showing a four-fold increase in ectopic Rab5 levels with respect to control cells, confirming the data observed by time-lapse videomicroscopy. Notably, Rab5-stained endosomes accumulate in control cells treated with the calpain inhibitory peptide, calpeptine, just like upon CAPNS1 depletion (Fig. S1). Collectively, these results demonstrate that calpain deficiency affects early endosome dynamics, upon thapsigargin treatment. The accumulation of both RFP-GFP-LC3 and GFP-Rab5 in CAPNS1-depleted cells as compared with control cells, argues for a role of CAPNS1 in the early stages of autophagy.

### CAPNS1 depletion is coupled to Golgi stack redistribution

**Fig. 4.**
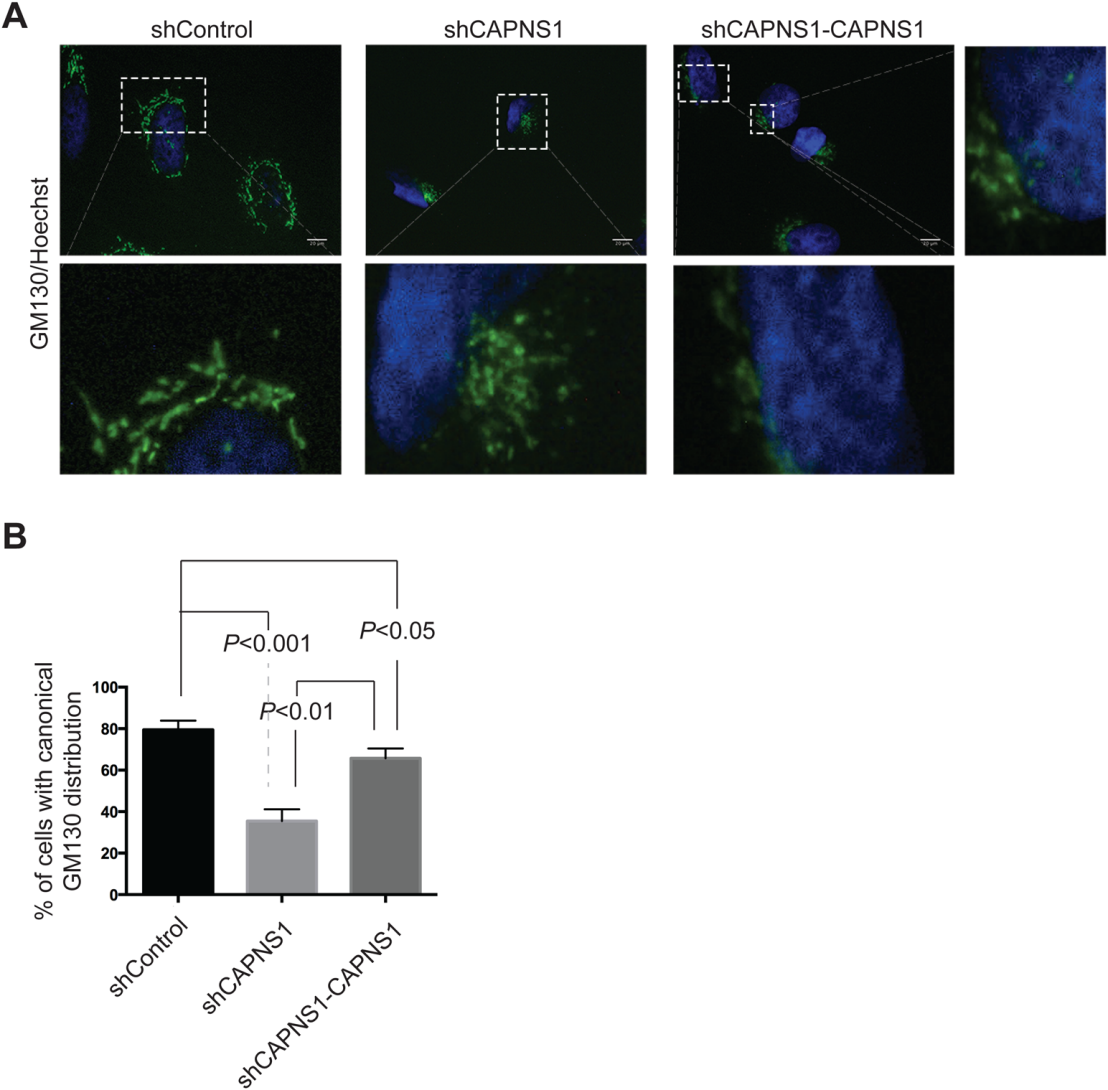
**CAPNS1 depletion perturbs Golgi stack distribution.** (A) Control, shCAPNS1 and CAPNS1-rescued U2OS cells were fixed and stained with GM130 antibody. Hoechst dye was used to stain nuclei. A fluorescence microscope was used to acquire pictures; Scale bars: 20 μm. Lower and right panels are magnifications of boxed areas. 100 cells for each sample were considered and the percentage of cells with Golgi stacks surrounding the nucleus was quantified. (B) Means and s.d. of three independent experiments.

The Golgi network represents one of the membrane sources for the formation and elongation of the phagophore, together with the ER and mitochondria (Matsunaga et al., 2010; Ylä-Anttila et al., 2009; Hailey et al., 2010; Hamasaki et al., 2013). In order to monitor the effect of CAPNS1 depletion on Golgi architecture, the endogenous cis-Golgi protein GM130 was analysed by immunostaining in control U2OS cells, CAPNS1-depleted cells, and their derivatives with reintroduced CAPNS1. Fluorescence analysis reveals that in CAPNS1-depleted U2OS cells the Golgi network is condensed at one side of the nucleus, as compared with control cells, where its distribution is more scattered. Both distributions are observed in CAPNS1-rescued cells (Fig. 4A). The graph in Fig. 4B shows the average percentage of cells with a canonical Golgi stack distribution. Accordingly, in mouse embryonic fibroblasts, CAPNS1 depletion is also coupled to a perturbation in GM130 localization. Indeed, GM130-positive stacks are more tightly clustered near the nucleus (Fig. S2A). The Golgi apparatus was also analysed by transmission electron microscopy. In control cells, Golgi stacks surround the nucleus, while in CAPNS1-depleted cells, the Golgi network is mainly gathered at one side of it, confirming the immunofluorescence data (Fig. S2B). Altogether, these results indicate that calpain activity is important to regulate Golgi apparatus distribution. A disruption of the regular localization of Golgi stacks within the cytoplasm might also have consequences on the autophagic process.

### CAPNS1 depletion is coupled to impairment of Atg9 traffic

**Fig. 5.**
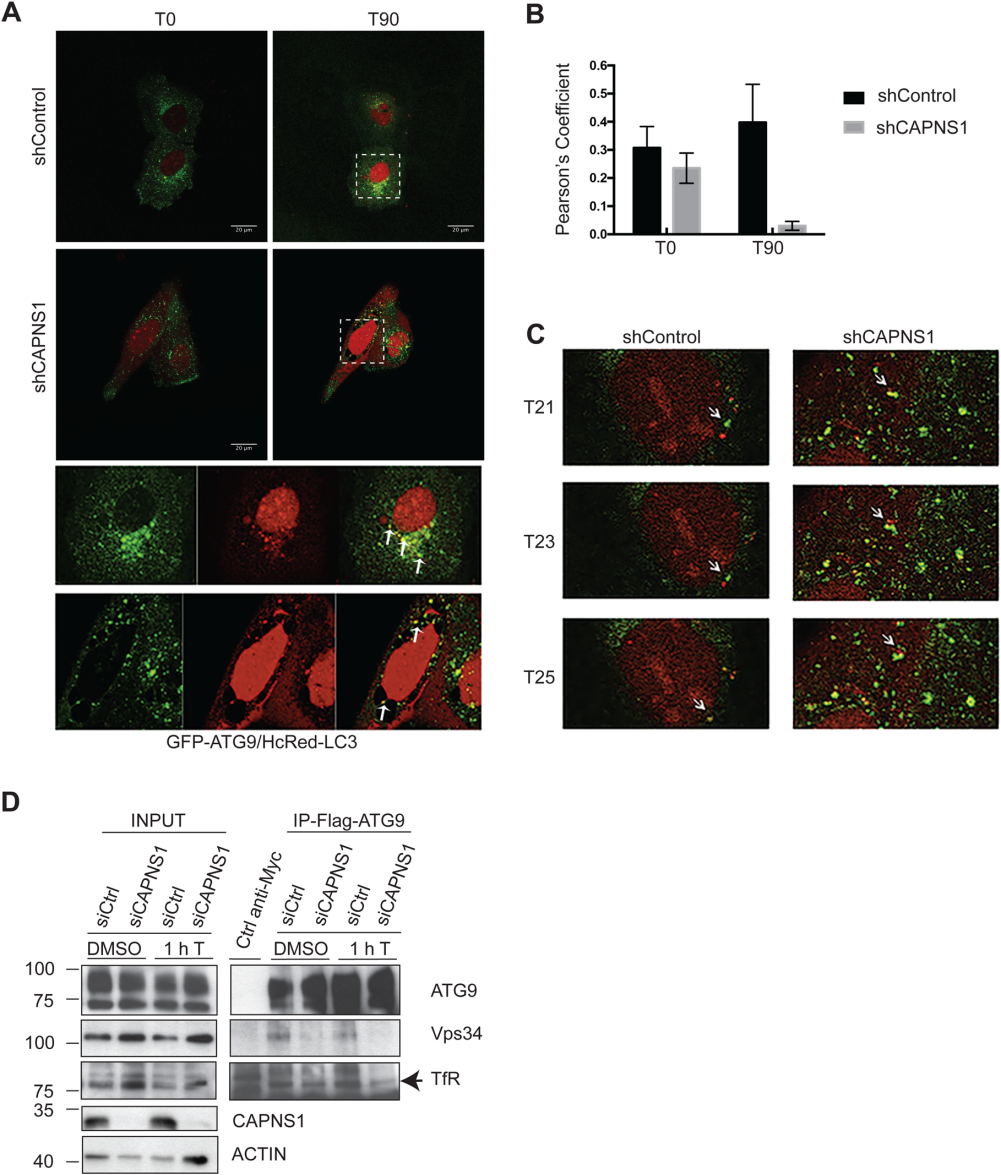
**Atg9 dynamics is impaired in CAPNS1-depleted cells.** (A) ShCAPNS1 and control cells were co-transfected with GFP-Atg9 and HcRed-LC3. 24 h later, the cells were analysed using a confocal microscope. Images were acquired every 2 min over a 50 min time interval. The first (T0) and last (T90) merged pictures of the 2 h time-lapse experiments are shown. Scale bars: 20 μm. Magnifications of boxed areas are shown in the right panels. Arrows indicate ATG9 vesicles adjacent to or fused with LC3 vesicles. (B) Quantification of GFP-Atg9 and HcRed-LC3 colocalization by Pearson's coefficient determination in control and CAPNS1-depleted U2OS cells, before and after thapsigargin treatment. (C) Images sequences (T21, T23, T25) of the GFP-ATG9/HcRed-LC3 time-lapse experiment made using control and CAPNS1-depleted U2OS cells. The arrow indicates one ATG9 vesicle that meets and fuses with a LC3-positive vesicle in control cells. In shCAPNS1 cells, ATG9- and LC3-positive vesicles are more stationary. (D) U2OS cells stably expressing Flag-Atg9 were transfected with a CAPNS1-specific siRNA or a control siRNA. 48 h later, the cells were treated for 1 h with DMSO or 100 nM thapsigargin before lysis and immunoprecipitation with an anti-Flag antibody. The immunoprecipitation products were analysed by western blot to visualize Flag-Atg9, endogenous TfR and Vps34.

We hypothesized that calpain could regulate the delivery of membranes toward the site of autophagosome formation. Atg9 is the only transmembrane autophagic protein. Atg9 is localized in the Golgi apparatus and late endosome vesicles. Upon autophagy induction, it re-distributes in the peripheral cytoplasm where it co-localizes with LC3 (Young et al., 2006). Since calpain depletion affects both dynamics and Golgi stack distribution of LC3 bodies, we investigated Atg9 and LC3 dynamics in our cellular system. Control and shCAPNS1-depleted U2OS cells were transiently co-transfected with GFP-Atg9 and HcRed-LC3 expression plasmids. 24 h later, the cells were analysed by *in vivo* imaging in a 90 min time-lapse experiment. After the first 15 min of image acquisition, 100 nM thapsigargin was added to induce autophagy (Movies 5,6). In control cells, Atg9 clearly localizes in a perinuclear region (Fig. 5A). After autophagy induction, Atg9 vesicles freely move and eventually fuse to LC3 dots (Fig. 5C). On the contrary, in shCAPNS1 cells, Atg9-positive vesicles are more stationary, they may stay in close proximity to LC3 bodies, but do not fuse with them (Fig. 5C). The degree of co-localization between HcRed-LC3-positive vacuoles and GFP-Atg9-positive vacuoles was quantified measuring the Pearson's coefficient of ≥10 cells under each condition in three independent experiments (Fig. 5B). Notably, unstained vesicles appear near the nucleus (Fig. 5A, right panels). A very similar LC3 staining was previously observed in HCT-116 cells treated with cyclopentenone prostaglandin derivative (Kar et al., 2009). To further assess the involvement of calpain in Atg9 vesicles dynamics, we followed a biochemical approach and investigated Atg9 interaction with TfR and Vps34 in presence or absence of CAPNS1. U2OS cells, stably expressing Flag-Atg9, were used to transiently deplete CAPNS1. CAPNS1-depleted and control cells were treated or not with 100 nM thapsigargin. One hour later, the cells were lysed and used to immunoprecipitate Flag-Atg9. Confirming published data, Atg9 interacts with TfR in control cells. Notably, in CAPNS1-depleted cells this interaction is less evident, and it is further reduced upon autophagy induction with 100 nM thapsigargin (Fig. 5D). In control cells, Atg9 interacts with Vps34, a component of the class III PI3K complex, underlining the importance of Atg9 in the initial steps of the autophagic process (Fig. 5C). Notably, VPS34 does not co-immunoprecipitate with Atg9 in CAPNS1-depleted cell lysates. Taken together, these data indicate that calpain allows trafficking of Atg9 toward the nascent autophagosome.

### CAPNS1 depletion prevents trafficking of Atg9- and Bif-1-containing vesicles upon autophagy induction by thapsigargin

**Fig. 6.**
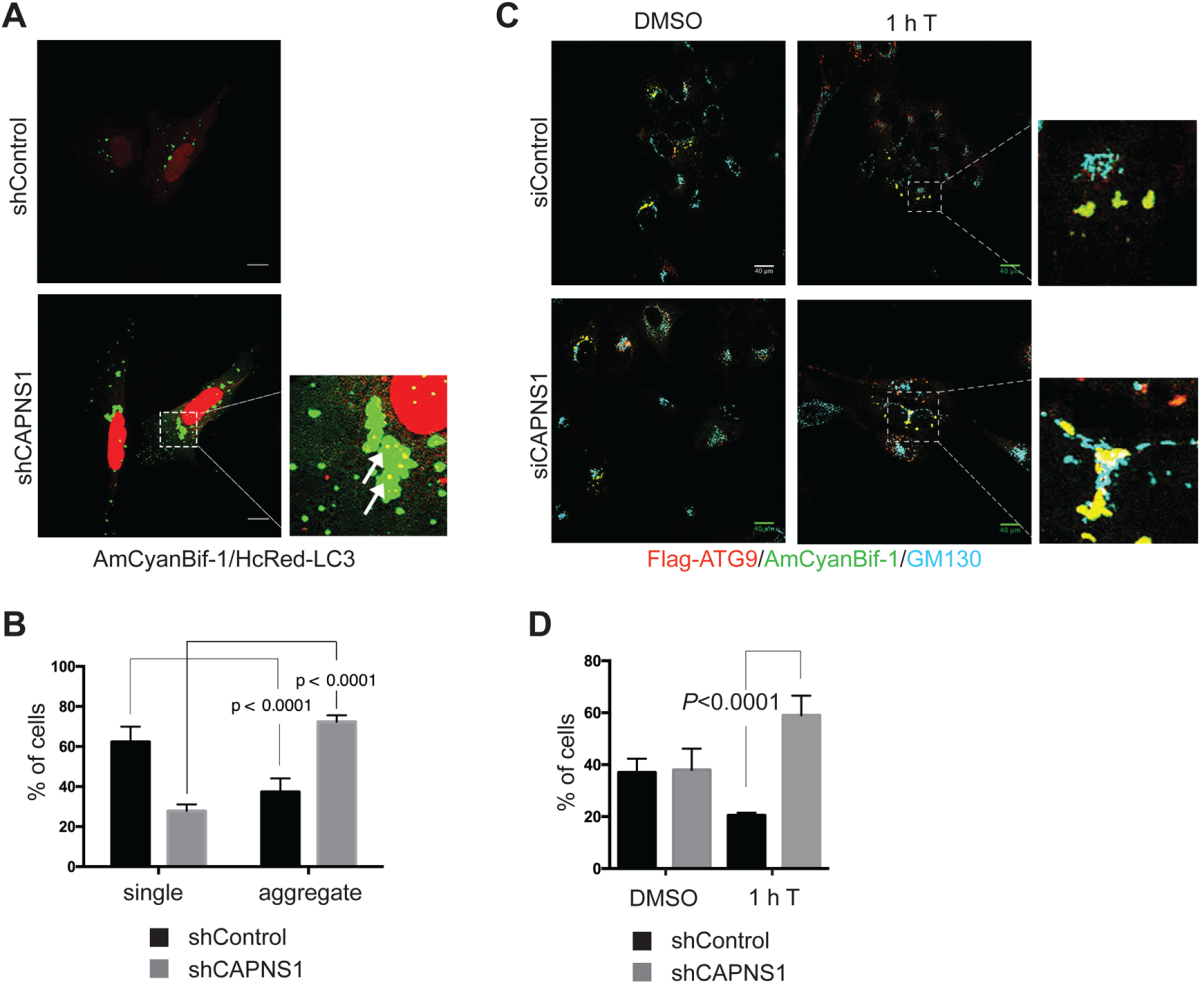
**Atg9/Bif-1 trafficking from the Golgi is impaired in CAPNS1-depleted cells.** (A) Control and shCAPNS1 U2OS cells were transiently co-transfected with AmCyan-Bif-1 and HcRed-LC3. 16 h later, the cells were fixed and analysed by a confocal microscope. Arrows indicate areas of colocalization of AmCyan-Bif-1 and HcRed-LC3. Scale bars: 20 μm. (B) 100 cells for each sample were considered and the number of cells where Bif-1 forms aggregates or single dots was counted. The graph represents the means and s.d. of three independent experiments. (C) U2OS cells stably expressing Flag-Atg9 were transiently silenced with control or CAPNS1-specific siRNA and then transfected with AmCyan-Bif-1. 24 h later the cells were treated for 1 h with or without 100 nM thapsigargin, and then fixed and analysed by immunofluorescence to visualize endogenous GM130, Flag-Atg9 and AmCyan-Bif1. Representative confocal merged images are shown. Scale bars: 20 μm. (D) 50 cells for each sample were considered and the number of cells where Atg9/Bif-1 positive vesicles colocalize with endogenous GM130 was counted. Mean and s.d. of three independent experiments.

Bif-1 interacts directly with the double lipid layer of membranes, trough its N-BAR domain, promoting Golgi tubule fission and delivery of Atg9 vesicles to the nascent autophagosome upon autophagy induction (Takahashi et al., 2011). As a first approach to evaluate any effect of calpain on Bif-1, we checked its distribution with respect to LC3, in the presence or absence of CAPNS1. AmCyan-Bif1- and HcRed-LC3-expressing constructs were transiently transfected in control and CAPNS1-depleted U2OS cells. As shown in Fig. 6A,B, CAPNS1 depletion is coupled to an accumulation of Bif-1 positive aggregates near the nucleus, when compared with control cells.

In order to study the effect of CAPNS1 depletion on Atg9 and Bif-1 trafficking, we utilized a U2OS cell line stably expressing Flag-Atg9 and transiently transfected with AmCyan-Bif-1-expressing plasmid. CAPNS1-specific or control siRNA were transiently transfected in this system and the cells were treated or not for 1 h with 100 nM thapsigargin to induce autophagy. Flag-Atg9, AmCyan-Bif-1, and endogenous GM130 were analysed by fluorescence microscopy. Upon thapsigargin treatment, Bif-1/Atg9 double-positive vesicles move away from endogenous GM130 in control cells (Fig. 6C, top panels). On the contrary, in CAPNS1-depleted cells, Bif-1 and Atg9 remain on GM-130 positive Golgi membranes (Fig. 6C, bottom panels). The graph in Fig. 6D indicates the average number of cells where Flag-Atg9/AmCyan-Bif-1 double-positive vesicles are stained also by anti-GM130 antibody. Collectively, these data indicate that calpain allows the fission of Bif-1/Atg9 vesicles from the Golgi apparatus.

### Bif-1 protein is cleaved by calpain

**Fig. 7.**
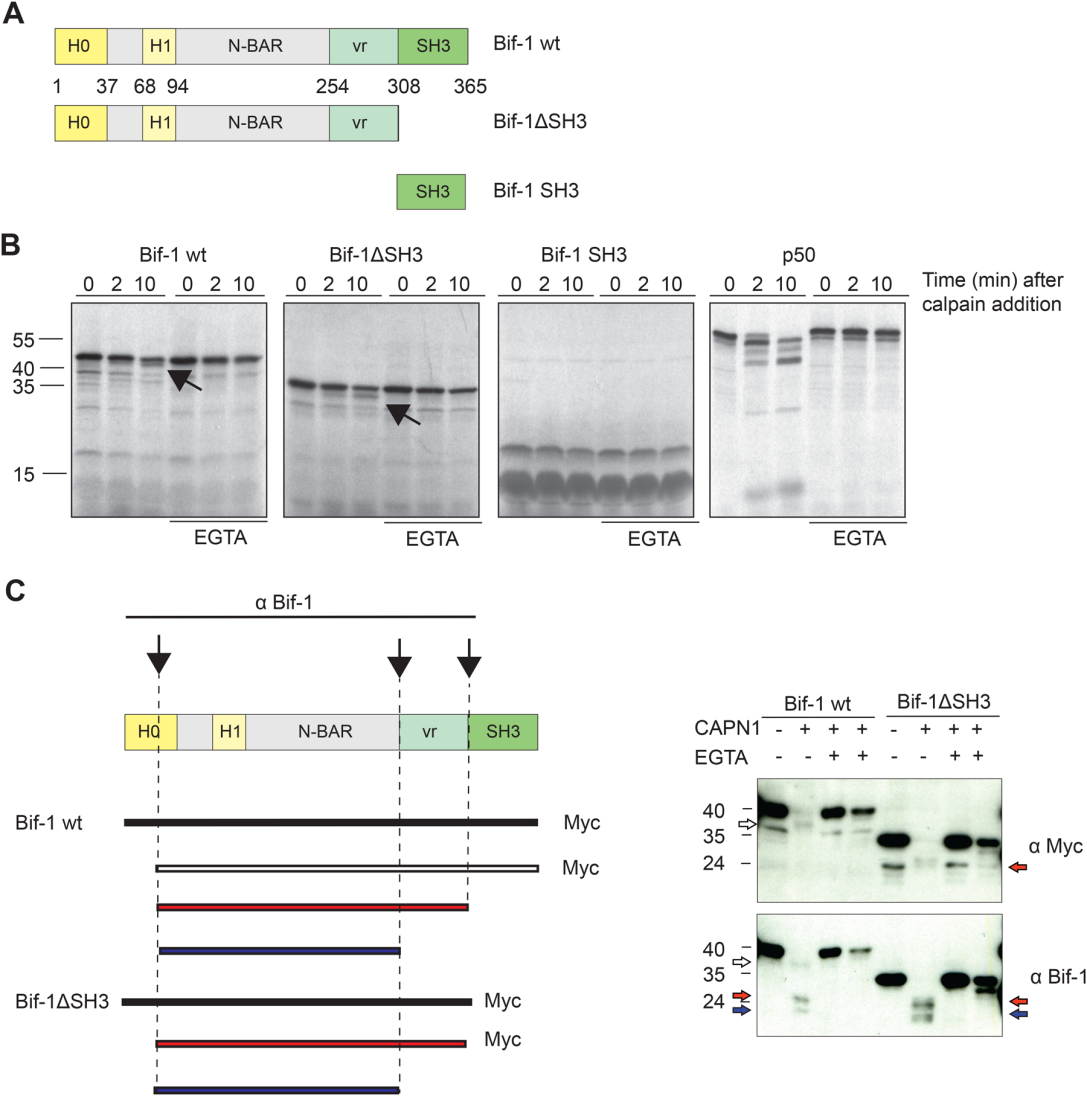
**The N-terminal region of Bif-1 protein is cleaved by calpain.** (A) Schematic representation of the structure of wild-type Bif-1 and mutants. Bif-1 contains two domains involved in membrane binding: H0 and H1, a N-BAR domain and a C-terminal SH3 domain. (B) Bif-1 wt, Bif-1 ΔSH3, Bif-1 SH3 and p50 NF-κB1 were produced as ^35^S-methionine-labeled proteins by *in vitro* transcription and translation and incubated for the indicated time intervals with commercial micro-calpain, as previously described (Demarchi et al., 2005). The reactions were then stopped in Laemmli buffer and analysed by SDS-PAGE and autoradiography. Parallel reactions in the presence of 10 μM EGTA were carried out to prove the calcium dependency of the reactions. Arrows indicate calpain cleavage products. (C) Schematic drawing of Bif-1 and its derivative fragments obtained after calpain cleavage and analysed by western blot anti-myc tag and anti-Bif-1 reported on the right side of the panel. The white arrow indicates the wt Bif-1 cleavage product retaining the SH3 domain. The solid arrows indicate lower molecular weight cleavage products lacking the C-terminal SH3 domain.

By means of bio-informatics tools based on the Multiple Kernel Learning algorithms, it is possible to identify putative calpain cleavage sites on an amino acid sequence (DuVerle et al., 2011). The two sites with the highest calpain cleavage score on Bif-1 are located between the BAR domain and the SH3 domain at amino acids 296 and 290. Notably, potential calpain cleavage sites are present also on Atg9 protein sequence. To verify the predicted cleavage sites on Bif-1, we performed an *in vitro* calpain cleavage assay. Wild-type Bif-1, Bif-1 lacking the SH3 domain, and the SH3 domain of Bif-1 (Fig. 7A) were produced as [^35^S]-methionine-labelled proteins by *in vitro* transcription and translation. NF-κB1 p50 was produced as a positive and negative control for the cleavage experiment. The radioactive products were incubated for 0, 2 and 10 min with commercial micro-calpain. As indicated by the arrows in Fig. 7B, a cleavage products originates both from Bif-1 wild-type (wt) and Bif-1 ΔSH3, but not from Bif-1 SH3. EGTA, an inhibitor of calpain, prevents Bif-1 digestion. These results demonstrate that micro-calpain cuts Bif-1 outside the SH3 domain. Moreover, since after calpain cleavage, the decrease in size of both full-length and Bif-1-ΔSH3 is comparable, we predict that the cleavage occurs near the N-terminal end of the protein. In order to test this hypothesis, wt Bif-1-myc and Bif-1-ΔSH3-myc at 0 and 10 min after calpain digestion and respective EGTA containing controls were separated on gel and analysed by immunoblot using anti-myc tag or anti-Bif-1 antibodies (Fig. 7C). The anti-myc antibody recognizes a digestion product of the same molecular mass of the fragment visualized by autoradiography (white arrow). On the other hand, the anti-Bif-1 antibody detects also two lower molecular mass bands (red and blue arrows). On the basis of their molecular mass, we speculate they could derive from subsequent processing that remove the SH3 domain or both the SH3 domain, and the variable region. This hypothesis is schematized in the cartoon of Fig. 7C. *In vitro* calpain cleavage assays using GST-Bif-1 as a substrate, further suggest that a first cleavage occurs at the very N-terminal end of Bif-1 (Fig. S3). We failed to detect any cleavage product of endogenous Bif-1 by analysing total cellular lysates by western blot. We hypothesize that the cleavage products have a very short life within the cell, as described for other calpain targets, thus allowing a tightly controlled and transient activity of the active protein.

### Overexpression of E28A Bif-1 is coupled to autophagic block

**Fig. 8.**
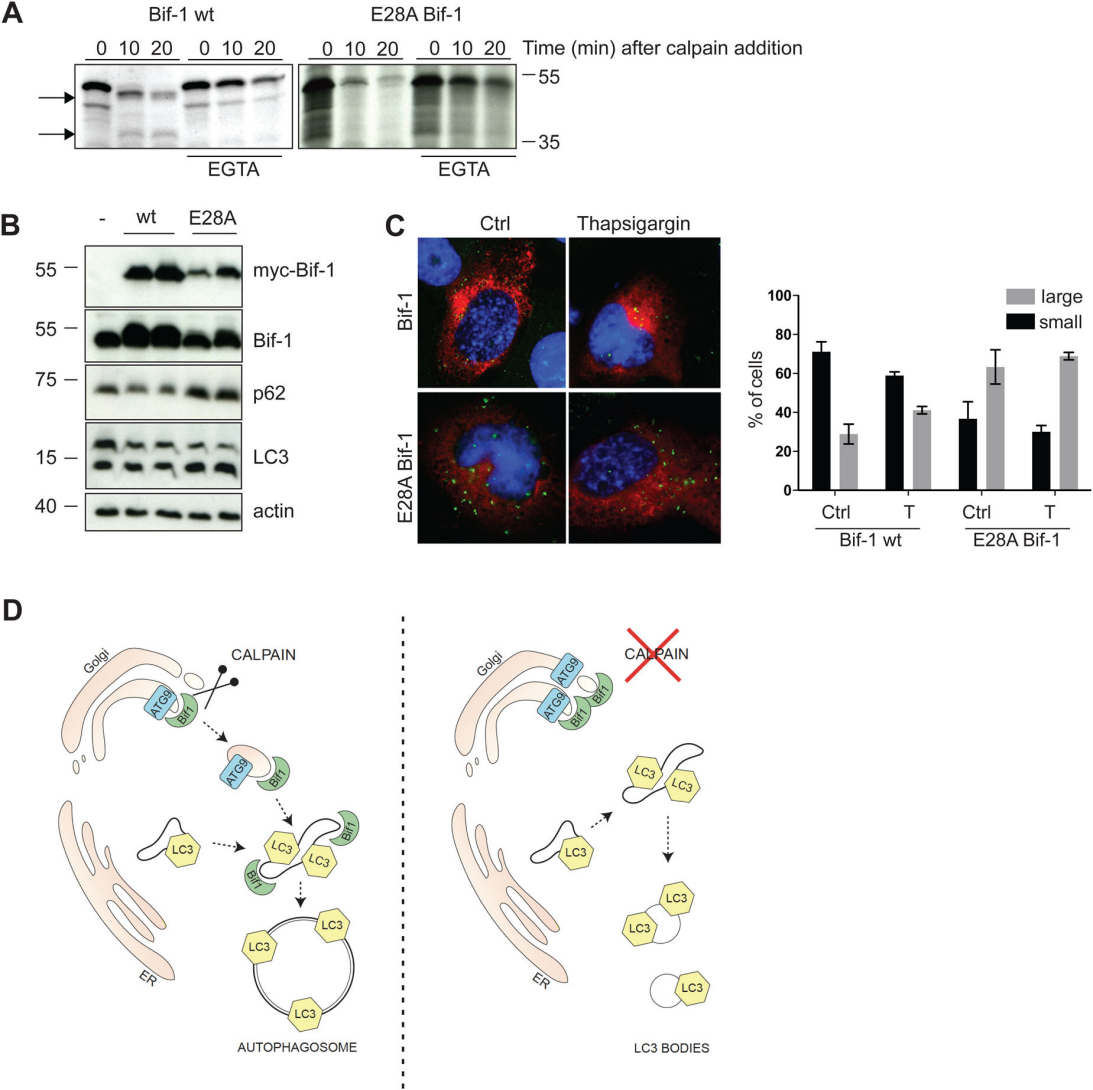
**Amino acid E28 on Bif-1 is important for calpain-mediated processing and autophagic clearance of p62.** (A) Wild-type Bif-1 and E28A Bif-1 were produced as ^35^S-methionine-labeled proteins by *in vitro* transcription and translation and incubated for the indicated time intervals with commercial micro-calpain, as previously described (Demarchi et al., 2005). The reactions were then stopped in Laemmli buffer and analysed by SDS-PAGE and autoradiography. Parallel reactions in the presence of 10 μM EGTA were carried out to prove the calcium dependency of the reactions. Arrows indicate calpain cleavage products. (B) The myc-tagged constructs: wild-type Bif-1 and E28A Bif-1 were transfected into H1299 cells. 24 h later, the cells were treated or not with 100 nM thapsigargin for 1 h. Afterwards, the cell lysates were prepared and analysed by western blot with the indicated antibodies. (C) The myc-tagged constructs: wild-type Bif-1, and E28A Bif-1 were transfected into H1299 cells. 24 h later, the cells were treated or not with 100 nM thapsigargin for 1 h. Afterwards, the cells were fixed and analysed by immunofluorescence using anti-myc and anti-LC3 antibodies. Myc is stained in red, endogenous LC3 is stained in green. Scale bars: 20 μm. Graph shows the percentage of cells with small/large LC3 dots. The error bars represent s.d. of three different independent experiments. At least 25 cells were analysed for each replicate. (D) Working model. Calpain processes Bif-1, and allows delivery of Atg9-Bif-1 vesicles to the nascent autophagosome. In the absence of calpain, LC3-positive bodies accumulate in the cells.

Using the freely available software described by the group of Sorimachi (DuVerle et al. 2011), we searched for cleavage sites in the first 37 amino acids, corresponding to the first domain of Bif-1, which is required for membrane binding. By means of this bioinformatics tool, we found that glutamic acid 29 is the best candidate for calpain cleavage. Therefore, we produced one E28A Bif-1 point mutant. We selected amino acid 28 since it corresponds to position P2 respect to the putative cleavage site at lysine 29 identified by in silico analysis, and is therefore essential for site selection. As shown in Fig. 8A, the mutant is resistant to calpain proteolytic processing in the N-terminal region. Notably, the mutant protein is completely degraded by calpain *in vitro*. This result suggests that, upon loss of the preferential processing site, calpain can cleave Bif-1 at several sites, originating unstable peptides. In order to investigate whether overexpression of E28A Bif-1 might impact autophagy in the cell, even in the presence of endogenous Bif-1, we employed a cell line characterized by a high level of basal autophagic flux. In particular, we selected H1299, a non small cell lung carcinoma cell line. E28A Bif-1-myc and wild-type Bif-1-myc constructs were transfected in H1299 cells and 24 h later, incubated or not with thapsigargin for 1 h. Next, cellular lysates were prepared and analysed by immunoblot. As shown in Fig. 8B, overexpression of each point mutant leads to a sharp accumulation of both p62, and LC3-II, suggestive of impaired autophagy. Thapsigargin treatment in this cell line does not further increase LC3 lipidation, possibly because the autophagic flux is already intense in this cell line. Indeed, as shown in the representative images of Fig. 8C, endogenous LC3 dots, stained in green, are already present in basal conditions, confirming the high autophagic activity of the H1299 cell line. Notably, endogenous LC3 dots are more numerous and appear to form larger structures in the cells overexpressing E28A Bif-1. Altogether, these results demonstrate that a point mutation of Bif-1 at glutamic acid 28 results in the impairment of calpain mediated processing of Bif-1 N-terminal domain. Furthermore, overexpression of E28A Bif-1 in H1299 cells is coupled to the accumulation of p62 and LC3 aggregates.

## DISCUSSION

A large body of studies indicate that in mammalian cells the endoplasmic reticulum is a site for autophagosome nucleation (Hamasaki, 2013). Moreover, the endocytic network contributes to phagophore formation and expansion as well as autophagosome maturation (Moreau et al., 2011; Lamb et al., 2013). Notably, Atg9, the only known transmembrane autophagic protein is present both in the endosomal compartment, and Golgi apparatus, both described as autophagosome membrane sources (Orsi et al., 2012; Puri et al., 2013). The fission of Atg9-containing vesicles from the Golgi stacks involves the endophilin Bif-1, which also plays a critical role in vesicle formation for coat protein I (COPI)-mediated retrograde transportation from the trans-Golgi network to the endoplasmic reticulum (Yang et al., 2006). Here, we describe the requirement of CAPNS1 for the trafficking of Atg9/Bif-1-bearing vesicles from the Golgi apparatus, and for the interaction of Atg9 with the autophagic essential kinase Vps34. The fusion of Bif-1/Atg9-containing vesicles with LC3 bodies budding from the ER, may allow membrane bending and formation of the mature double membrane autophagosome (Takahashi et al., 2007, 2011). It is well established that calpain-mediated processing can confer novel functions to their substrates (Sorimachi et al., 2011). Our study identified a calpain cleavage site on the N-terminal region of Bif-1. We hypothesize that calpain-mediated processing of Bif-1 N-terminus may remove the anchorage that retains outgoing vesicles in the Golgi apparatus. Therefore, this cut may be instrumental for the fission of tubular elements containing Atg9 from the Golgi apparatus, and subsequent fusion with LC3 vesicles coming from the endoplasmic reticulum. Interestingly, in macrophages, LC3-associated phagocytosis was described as a mechanism involving many autophagic players, but without the formation of a double membrane (Martinez et al., 2015). Possibly, a similar pathway may exist also in other cell types allowing an alternative way for membrane trafficking involving LC3. We also found other potential calpain cleavage sites by our *in vitro* studies. We speculate that subsequent cleavages that remove the SH3 domain may allow the removal of Bif-1 and its binding partners from the mature autophagosome. An alternative mechanism for Bif-1 regulation by calpain was proposed by Wong and colleagues (Ganley et al., 2011). They studied Bif-1-dependent autophagy induction in neurons. According to their study, calpain might cleave p35 to release p25, which activates Cdk5 and this in turn phosphorylates Bif-1 at T145, which is a prerequisite for proper Bif-1 activity. Possibly, both calpain-dependent CDK5-mediated phosphorylation and calpain-mediated processing may occur sequentially, as reported for talin modulation.

Time-lapse analysis of CAPNS1-depleted U2OS cells reveals an LC3 staining coupled to cytoplasmic vacuolation upon thapsigargin treatment. A very similar pattern was previously observed by others in HCT-116 upon cyclopentone prostaglandin derivative treatment (Kar et al., 2009). The vacuolation was reported to be a consequence of ER stress-induced ER dilation, subsequently leading to cell death. In our study too, vacuolation is coupled to ER stress induced by thapsigargin. Moreover, we previously reported increased cell death in CAPNS1-depleted cells upon damage/stress (Demarchi et al., 2006).

We observed an enlargement of the endosome compartment upon thapsigargin treatment in CAPNS1-depleted cells. Interestingly, enlarged vesicles containing the Rab5 effector EEA1 were described in Bif-1-depleted cells (Wan et al., 2008). This similar phenotype may suggest that calpain is also important for Bif-1 role in endosome trafficking and function. Notably, a protein complex consisting of TIP30, Bif-1 and ACSL4 is crucial for moving Rab5 and v-ATPase to endosome precursors (Zhang et al., 2011).

Other studies indicate a negative role for calpain in the autophagic process (Menzies et al., 2015), but this is not surprising, given the pleiotropic functions of these processing proteases and the tightly regulated transient activation of these enzymes. Indeed, calpain was reported to have both positive and negative roles in cellular movement. As far as autophagy is concerned, calpain may exert the cleavage of Bif-1 to allow scission of Golgi components and their targeting to the nascent autophagosomes. Subsequently, or in presence of excessive stress, it may cleave essential proteins such as Atg5 (Yousefi et al., 2006) and switch off the process. We propose that upon transient activation of calpain in response to cellular stressors, Bif-1/Atg9-bearing vesicles move from the Golgi network toward the site of autophagosome formation, where they interact with Class III PI3K Vps34. Subsequently, Atg9 is recycled through the endocytic pathway, and it interacts with the transferrin receptor. When the cellular stress becomes overwhelming, calpain hyperactivation leads to inhibition of autophagic players and to calpain inhibition by calpastatin. At this point, an alternative pathway involving LC3 bodies becomes prevalent over macroautophagy.

## MATERIALS AND METHODS

### Plasmids and reagents

The pAmCyan-N1-Bif1, pGEX-4T1-Bif-1, pEF6-Bif-1wt-Myc-HisA, pEF6-Bif-1dSH3-Myc and the pEF6-Myc-HisA-Bif-1S were a kind gift from Dr Hong-Gang Wang (Penn State College of Medicine, Hershey, PA, USA) pHAGE-N-GFP-ATG9 and MSCV-Tet-FLAG-HA-IRES-PURO-ATG9 were generously provided by Dr Ivan Dikic (Institute of Biochemistry II and BMLS Goethe University School of Medicine, Frankfurt, Germany). Thapsigargin and IPTG were from Sigma. Indo1-AM was purchased from Thermo Fisher Scientific. Antibodies were obtained from the following sources: rabbit anti-ATG9 (Cat. NBP195342, Clone 9B11); mouse anti-Bif-1 (Cat. NBP2-24733, Clone 30A882.1.1); and rabbit anti-pIRE (Cat. NB1002323); from Novus Biologicals; mouse anti-Flag (Cat. F3165, Clone M2), rabbit anti-Actin (Cat. A2066), and mouse anti-CAPNS1(Cat. C0230, Clone 28F3) from Sigma; mouse anti-GM130 (Cat. 610822, Clone 35) and mouse anti-p62 (Cat. 610832, Clone 3) from BD Transduction Laboratories; mouse anti-Myc-Tag (Cat. 2276, Clone 9B11) from Cell Signaling; goat anti-CAPN1 precursor (sc-7531, Clone N-19) and goat anti-CD71(TfR) (sc-32272, Clone 3B82A1) from Santa Cruz; rabbit anti-active CAPN1 (ab28257) from Abcam; rabbit anti-Vps34 (Cat. 382100) from Thermo Fisher Scientific. LC3 antibodies were purified from rabbit serum after immunization with GST-LC3 according to standard procedures. siRNA targeting CAPNS1 was purchased from Eurofins MWG Operon (Germany): the pool of four siRNAs targeting CAPNS1 (1-GAG CAU CUC UAU AAC AUG AUU TT, 2-CCA CAG AAC UCA UGA ACA UUU TT, 3-UCA GGG ACC AUU UGC AGU AUU TT, 4-GAA GAU GGA UUU UGA CAA CUU TT). The Baculovirus reagents, RFP-GFP-LC3, GFP-Rab5a and GFP-E1-alpha-pyrivate-dehydrogenase, were purchased from Thermo Fisher Scientific. For the *in vitro* transcription and translation of radio-labelled proteins TnT T7 Quick Coupled Transcription/Translation System (Promega) was used.

### Cell culture, transfection and shRNA-mediated gene silencing

All the cells are routinely checked for contamination. U2OS cells were obtained from ATCC and recently authenticated. They were grown in Dulbecco's modified Eagle's medium (DMEM) low glucose, supplemented with 10% FCS, 1% penicillin/streptomycin (Lonza) and L-glutamine. U2OS pRS-control and pRS-shCAPNS1 (Raimondi et al., 2016) were produced according to standard procedures. For cell infections, 293GP packaging cells were transfected with the calcium-phosphate method with pRetroSuper-shCAPNS1 or vector alone, after 72 h the supernatant was harvested, filtered and added to U2OS cells. The infected cells were selected by the addition of puromycin and after 7 days the expression of CAPNS1 was checked by western blot. The same protocol was used to produce U2OS cells stably expressing HA-Flag-ATG9. For the production of the shCAPNS1 stable cell line with the reintroduction of calpain small subunit, the U2OS cells describe above were infected with a pwzL vector expressing CAPNS1. 293T cells were grown in DMEM high glucose, supplemented with 10% FCS and 1% penicillin/streptomycin. Wild-type, CAPNS1^−/−^ and rescued mouse embryonic fibroblasts (Arthur et al., 2000) were a kind gift of Dr Peter A. Greer (Queen's Cancer Research Institute, Ontario, Canada); cells were grown in DMEM high glucose, supplemented with 10% fetal calf serum (FCS), 1% penicillin/streptomycin and Non-Essential Amino acid solutions 100× (Sigma). For transient transfection and silencing, TransIT-LT1 transfection reagent (Mirus) and Lipofectamine RNAiMAX (Invitrogen) were used respectively, according to the manufacturer's instructions.

### Kinetic analysis of intracellular calcium concentration

U2OS cells were incubated for 30 min at 37°C with 2.5 μM Indo1-AM in DMEM-1%FCS. Next, the cells were washed and incubated in DMEM-1%FCS for 20 min at room temperature. The samples were then run through the flow cytometer BD LSRFortessa Analyzer for 3 min and then for additional 4 min after thapsigargin (100 nM) addition. FlowJo software version 8.8.7 was used to analyse the data. Indo-1 emission peak shifts from 485 nm (Indo Blue) for unbound dye to 405 nm (Indo Violet) when the Indo1 molecule is bound to calcium. Mean intracellular calcium concentration is quantified in terms of the ratio of 405 nm/485 nm Indo1 emission peaks. Moving average was applied to increase the signal to noise ratio, as previously described (MacFarlane et al., 2010).

### Western blot analysis and immunoprecipitation

Cell lysates were obtained in 50 mM Tris-HCl pH 7.5, 150 mM NaCl, 5 mM EDTA, 1% Triton X-100, supplemented with 0.5 mM NaF, 2 mM EGTA, 1 mM sodium orthovanadate and complete protease inhibitor cocktail (Sigma). Lysates were clarified by centrifugation for 10 min at 4°C and protein concentrations were assessed using Bradford protein assay (BioRad Laboratories). Samples containing equal amounts of proteins were boiled in SDS sample buffer, resolved using SDS-PAGE and transferred to nitrocellulose membranes. The blots were then probed with the appropriate antibodies. Before immunoprecipitation, U2OS cells stably expressing HA-Flag-ATG9 were transiently silenced for CAPNS1 for 3 days, and then treated for 1 h with 100 nM thapsigargin. Whole cell lysates in 20 mM CHAPS, 125 mM NaCl, 50 mM Tris-HCl, pH 7.5, supplemented with 0.5 mM NaF, 1 mM Sodium orthovanadate, complete protease inhibitor cocktail (Sigma), were incubated for 2 h with anti-Flag antibody or with the anti-Myc antibody as negative control. Subsequently, protein G (GE Healthcare Life Sciences) was added for 2 h at 4°C. Samples were subjected to SDS-PAGE and immunoblotting.

### Confocal microscopy and live-cell imaging

U2OS cells transiently transfected with GFP- and AmCyan-tagged expression constructs or treated with commercial Baculovirus reagents were fixed on coverslips with 3% PFA for 20 min, washed with 0.1 M glycine in PBS and stained with Hoechst solution or propidium iodide for 5 min at room temperature. For immunofluorescence, after fixation with PFA, the cells were permeabilized with 0.1% Triton-X100 in PBS for 5 min, and blocked in PBS containing 5% BSA for 30 min at room temperature; then the slides were incubated with the appropriate antibodies (1:100) for 2 h at 37°C. Following 3 washings with PBS, samples were stained with secondary antibodies for 1 h at room temperature. Images were acquired with LSM510 confocal microscope (Zeiss), and processed using ImageJ software, freely available on the net. Imaging of live U2OS cells, grown on Nunc Glass Base dishes (Thermo Fisher Scientific) was performed on LSM510 confocal microscope (Zeiss), equipped with a cells incubator. Images were acquired every 2 min.

### Electron microscopy

Cells monolayers were fixed in 2% glutaraldehyde in 0.2 M Hepes buffer, pH 7.4, for 1 h at room temperature. Next the cells were incubated in 1% osmium tetroxide in 0.1 M sodium cacodylate buffer, pH 7.4, with the addition of 15 mg/ml of potassium ferrocyanide, for 1 h at room temperature. Then slides were dehydrated in a graded series of ethanol and embedded in Epon using routine procedures. Approximately 60 nm sections were cut and stained using uranyl acetate and lead citrate and were examined with a transmission electron microscope (Jeol JEM-1400).

### Expression and purification of GST-Bif-1

GST-Bif-1 was expressed by pGEX-4T-1 plasmid in BL21de3 strain of *Escherichia coli*. Briefly, transformed cells were grown in Luria Bertani medium containing 100 μg/ml ampicillin at 37°C to an A_600 nm_ 0.8; then 1 mM IPTG was added to induce protein expression at 37°C for 3 h. Cells were lysed in PBS, pH 7.4, supplemented with 0.5 mM NaF, 2 mM EGTA, 1 mM Sodium orthovanadate and complete protease inhibitor cocktail, by sonication and centrifuged at 8000 ***g*** for 30 min. The resulting supernatant was incubated with Glutathione Sepharose 4B (GE Healthcare) at 4°C for 1 h and then washed three times with PBS. The protein was eluted with 10 mM reduced glutathione in 50 mM Tris-HCl, pH 8.

### *In vitro* cleavage assay

^35^S-labelled *in-vitro* transcribed/translated proteins were produced by standard procedures and incubated with micro-calpain on ice in 10 mM Tris-HCl, pH 7.5, 1.5 mM DTT, 750 μM CaCl_2_ as described (Cataldo et al., 2013). Reactions were terminated at the indicated time points by adding SDS-PAGE loading buffer and analysed on SDS-PAGE*.*

### Statistical analysis

Results are expressed as means±s.d. of at least three independent experiments. Statistical analysis was performed using two-tailed Student's *t*-test or two-way ANOVA with the minimal level of significance set at *P*<0.05.
